# Corrigendum to “An inhibitor of interaction between the transcription factor NRF2 and the E3 ubiquitin ligase adapter β-TrCP delivers anti-inflammatory responses in mouse liver” [Redox Biol. 55 (2022) 102396/PMID: 35839629]

**DOI:** 10.1016/j.redox.2022.102428

**Published:** 2022-08-02

**Authors:** Raquel Fernández-Ginés, José Antonio Encinar, John D. Hayes, Baldo Oliva, Maria Isabel Rodríguez-Franco, Ana I. Rojo, Antonio Cuadrado

**Affiliations:** a Instituto de Investigaciones Biomédicas “Alberto Sols” UAM-CSIC, Instituto de Investigación Sanitaria La Paz (IdiPaz) and Department of Biochemistry, Faculty of Medicine, Autonomous University of Madrid, Madrid, Spain; b Centro de Investigación Biomédica en Red Sobre Enfermedades Neurodegenerativas (CIBERNED), ISCIII, Madrid, Spain; c Institute of Research, Development and Innovation in Biotechnology of Elche (IDiBE) and Molecular and Cell Biology Institute (IBMC), Miguel Hernández University (UMH), 03202, Elche, Alicante, Spain; d Jacqui Wood Cancer Centre, Division of Cellular Medicine, James Arrott Drive, Ninewells Hospital and Medical School, University of Dundee, Dundee, DD1 9SY, Scotland, United Kingdom; e Structural Bioinformatics Group (GRIB-IMIM), Department of Medicine and Life Sciences, Universitat Pompeu Fabra, Barcelona, Spain; f Instituto de Química Médica, Consejo Superior de Investigaciones Científicas (IQM-CSIC), C/ Juan de la Cierva 3, E-28006, Madrid, Spain

The authors regret that Martín Estrada-Valencia, who participated in the HPLC analysis of Fig. 8 was not included in the author list. The updated author list is as follows: Raquel Fernández-Ginés^1^, José Antonio Encinar^2^, John D. Hayes^3^, Baldo Oliva^4^, Martín Estrada-Valencia^5^, Maria Isabel Rodríguez-Franco^5^, Ana I. Rojo^1^, Antonio Cuadrado ^6^.

The authors would like to apologise for any inconvenience caused.

